# The role of oncogenic MUC1-C in brachyury-induced immunotherapy resistance

**DOI:** 10.1186/2051-1426-2-S3-P48

**Published:** 2014-11-06

**Authors:** Justin David, Duane Hamilton, Jeffrey Schlom, Claudia Palena

**Affiliations:** 1 Laboratory of Tumor Immunology and Biology, CCR, NCI, NIH, USA; 2 Laboratory of Tumor Immunology and Biology, CCR, NCI, NIH, Canada

## 

Epithelial-mesenchymal transition (EMT) is a molecular and cellular program in which epithelial cells lose their well-differentiated phenotype and adopt mesenchymal characteristics. This process occurs during the progression of cancer to metastatic disease, and is also associated with recurrence as conventional therapies fail to eliminate the minority of resistant tumor cells that have undergone EMT. Brachyury is a transcription factor that controls EMT during development, and we have previously reported that brachyury is aberrantly overexpressed in various human cancers where it promotes tumor cell EMT, metastatic dissemination, and resistance to conventional therapies. These studies led to the development of brachyury-based antitumor vaccine approaches that are currently in Phase I trials. However, we have recently shown that very high expression of brachyury can protect against caspase-dependent immune cell-mediated cytotoxicity [1]. In seeking to elucidate mechanisms of immunotherapy resistance, we have discovered a novel positive association between brachyury and the oncogenic cytoplasmic domain of mucin-1 (MUC1-C). MUC1-C is overexpressed in the majority of carcinomas, and it has been shown to confer resistance to genotoxic agents. We found that overexpression or silencing of brachyury in isogenic pairs and single-cell clones in colon, lung, pancreatic, and prostate cancer cell lines leads to increased or decreased MUC1-C mRNA and protein expression, respectively. We are currently investigating the association between brachyury and MUC1-C by immunohistochemical analysis of primary and metastatic tumor tissues. Because MUC1-C promotes resistance to cell death, we are also conducting experiments to ascertain whether inhibition of MUC1-C by 1) siRNA-mediated knockdown of MUC1-C, and 2) treatment with a MUC1-C inhibitor (currently in Phase I trials) reduces features of EMT, stemness, and resistance to immunotherapy in tumor cells that express very high levels of brachyury. Our ultimate goal is to demonstrate that MUC1-C inhibition can be combined with brachyury-based antitumor vaccine approaches to elicit potent immune responses against mesenchymalized cancer cells yielding durable tumor regression *in vivo*.
